# 

**Published:** 1893

**Authors:** 


					﻿Contents-
PAGE.
Hygiene Hints for Railway Travel.
Mrs. E. A. Matthews. ............ 193
Teplitz Mineral Water.
Robert Newman, M. D.............. 196
Mental Dyspepsia.
J. H. Kellogg,	M.	I)............ 200
The Chemistry of the Table.
Selected......................... 202
Miscellaneous Paragraphs........... ................ 204
House and Home. Conducted by Lillian White.......... 214
Editorial—Health is a Duty.......................... 219
Literary.........................................    220
General Noticps..................................... 224
				

